# Effects of Inactivated *Mycobacterium bovis* Vaccination on Molokai-Origin Wild Pigs Experimentally Infected with Virulent *M. bovis*

**DOI:** 10.3390/pathogens9030199

**Published:** 2020-03-07

**Authors:** Pauline Nol, Morgan E. Wehtje, Richard A. Bowen, Suelee Robbe-Austerman, Tyler C. Thacker, Kristina Lantz, Jack C. Rhyan, Laurie A. Baeten, Ramón A. Juste, Iker A. Sevilla, Christian Gortázar, Joaquín Vicente

**Affiliations:** 1 Centers for Epidemiology and Animal Health, Veterinary Services, Animal and Plant Health Inspection Service, United States Department of Agriculture, Fort Collins, CO 80526, USA; morganwehtje@gmail.com; 2 Dinosaur National Monument, Dinosaur, CO 81610, USA; 3 Department of Biomedical Sciences, Colorado State University, Fort Collins, CO 80523, USA; rbowen@rams.colostate.edu (R.A.B.); laurie.baeten@usda.gov (L.A.B.); 4 National Veterinary Services Laboratory, Veterinary Services, Animal and Plant Health Inspection Service, United States Department of Agriculture, Ames, IA 50010, USA; suelee.robbe-austerman@usda.gov (S.R.-A.); tyler.thacker@usda.gov (T.C.T.); kristina.lantz@usda.gov (K.L.); 5 National Veterinary Services Laboratory, Veterinary Services, Animal and Plant Health Inspection Service, United States Department of Agriculture, Fort Collins, CO 80521, USA; rhyanjack@yahoo.com; 6 National Wildlife Research Center, Wildlife Services, Animal and Plant Health Inspection Service, United States Department of Agriculture Fort Collins, CO 80521, USA; 7 Servicio Regional de Investigación y Desarrollo Agroalimentario SERIDA, Villaviciosa 33300, Asturias, Spain; 8 NEIKER-Instituto Vasco de Investigación y Desarrollo Agrario, Animal Health Department, Derio, 48160~Bizkaia, Spain; isevilla@neiker.eus; 9 SaBio Instituto de Investigación en Recursos Cinegéticos IREC, University Castilla la Mancha & CSIC, 13003 Ciudad Real, Spain; christian.gortazar@uclm.es (C.G.); joaquin.vicente@uclm.es (J.V.)

**Keywords:** *Sus scrofa*, wild pig, feral swine, *Mycobacterium bovis*, Mycobacterium tuberculosis Complex, tuberculosis, vaccination

## Abstract

The wild pig population on Molokai, Hawaii, USA is a possible reservoir for bovine tuberculosis, caused by *Mycobacterium bovis*, and has been implicated in decades past as the source of disease for the island’s domestic cattle. Heat-inactivated vaccines have been effective for reducing disease prevalence in wild boar in Spain and could prove useful for managing *M. bovis* in Molokai wild pigs. We designed an experiment to test this vaccine in wild pigs of Molokai genetics. Fifteen 3–4-month-old pigs were orally administered 10^6^–10^7^ colony forming units (cfu) of heat-inactivated *M. bovis* (Vaccinates; n = 8; 0.2 mL) or phosphate buffered saline (Controls; n = 7; 0.2 mL). Each dose was administered in a 0.5 mL tube embedded in a fruit candy/cracked corn mix. Boosters were given seven weeks post-prime in the same manner and dose. Nineteen weeks post-prime, pigs were orally challenged with 1 × 10^6^ cfu of virulent *M. bovis.* Twelve weeks post-challenge, pigs were euthanized and necropsied, at which time 23 different tissues from the head, thorax, and abdomen were collected and examined. Each tissue was assigned a lesion score. Ordinal lesion score data were analyzed using non-parametric Wilcoxon Signed Rank test. Effect size was calculated using *Cohen’s d.* Four of eight Vaccinates and four of seven Controls had gross and microscopic lesions, as well as culture-positive tissues. Vaccinates had statistically lower lesion scores than Controls in the following areas: gross thoracic lesion scores (*p* = 0.013 *Cohen’s d* = 0.33) and microscopic thoracic lesion scores (*p* = 0.002, *Cohen’s d* = 0.39). There were no differences in head lesion scores alone, both gross and microscopic, nor were there differences when comparing combined gross and microscopic head and thoracic lesion scores. These results are indicative that this vaccination protocol affords a modest degree of infection containment with this vaccine in Molokai wild pigs.

## 1. Introduction

Animal tuberculosis (TB), caused primarily by *Mycobacterium bovis*, a member of the *Mycobacterium tuberculosis* Complex (MTBC), is a globally significant disease that affects numerous livestock and wildlife species, as well as humans. Due to implementation in the United States of a national bovine tuberculosis eradication program over 100 years ago, TB prevalence has been reduced in cattle to less than 0.005% of herds today [1,2]. However, the presence of TB in free-ranging wildlife populations has impeded eradication efforts in the United States, as well as worldwide. In addition to the disease being endemic in white-tailed deer (*Odocoileus virginianus*) in the northeastern part of the state of Michigan, it is possible, but not established, that wild pigs (sometimes referred to as feral swine; *Sus scrofa*) on the Hawaiian island of Molokai may maintain the infection as well. *Mycobacterium bovis*-infected swine have periodically been detected on the island, and were thought to be the source of spillover to livestock in previous decades [3,4]. An effective vaccine against TB would be useful for managing *M. bovis* in the Molokai wild pig population, if the disease were determined to be endemic. Vaccination of free-ranging wildlife is feasible and has been shown to be successful in several wildlife species, including wild boar (*Sus scrofa*), red fox (*Vulpes vulpes*), and raccoon (*Procyon lotor*) [5,6,7]. Application of an orally-administered, heat-inactivated *M. bovis* vaccine to bovine tuberculosis-affected wild boar populations in Spain appears to have helped in reducing disease prevalence [8]. In addition, that vaccine preparation administered orally has shown some efficacy in protecting cattle and cervids, as well as goats and wild boar parenterally [9,10,11,12,13]. Here, we describe the use of heat-inactivated *M. bovis* vaccine in wild pigs of Molokai origin, and its efficacy against experimental infection with virulent *M. bovis*.

## 2. Results

### 2.1. Animals

No clinical signs or adverse effects were observed in any of the animals during the entire length of the experiment. All but one pig voluntarily consumed the bait after anesthesia recovery for prime vaccination. The exception was a pig that only successfully chewed and swallowed the bait once it was placed directly into its mouth. 

### 2.2. Gross and Microscopic Lesion Scores

Four of eight Vaccinates and four of seven Controls had visible gross lesions. Table 1 summarizes gross and microscopic lesion scores. 

**Figure 1:**
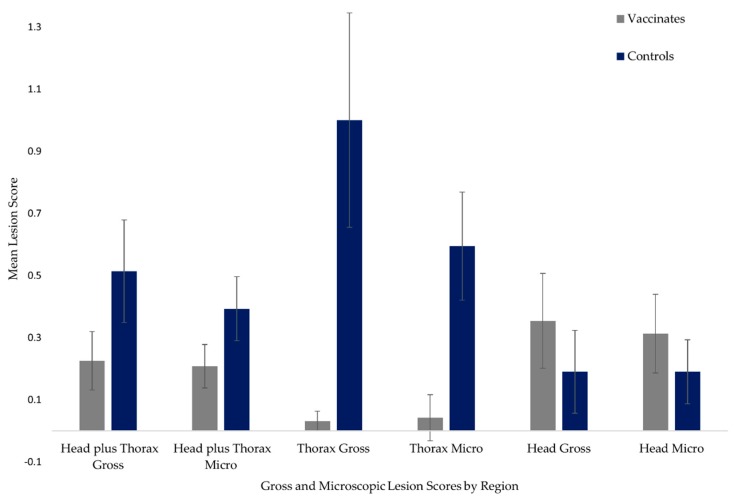
Mean gross and microscopic tissue lesion scores in Vaccinates and Controls by region. Means presented are for descriptive purposes only. Tissues collected at necropsy from Vaccinates and Controls 12 weeks post-challenge with 10^6^ colony forming units (cfu) virulent *M. bovis* strain 06-4387. Vaccinates received two oral doses of approximately 10^7^ cfu inactivated *M. bovis* at 19 and 12 weeks prior to challenge.

The total gross and microscopic lesion scores in Vaccinates were 18 and 21, respectively, while Controls had total gross and microscopic lesion scores of 36 and 33, respectively. Although means were not directly compared by statistical methods, mean gross and microscopic lesion scores associated with head and thoracic regions are presented in Figure 1 for descriptive purposes. There was no statistical difference and no effect between the treatment groups when taking into account combined head and thoracic gross lesion scores (p = 0.357, *Cohen’s d* = 0.08). The results were similar for combined microscopic lesion scores (p = 0.095, *Cohen’s d* = 0.14). In addition, there were no differences when comparing head region gross and microscopic lesion scores (p = 0.22 and 0.630, respectively). Neither head region group showed any effect size (*d* = 0.06 for gross and *d* = 0.13 for microscopic lesion scores). However, when comparing only thoracic scores, both gross and microscopic lesion scores were statistically lower in Vaccinates than in Controls (p = 0.013 and 0.002, respectively). *Cohen’s d* effect size was small to medium for these two lesion score groups (*d* = 0.33 and 0.40, respectively). There were no gross lesions visible in the abdominal region of either group, and only one microscopic lesion was detected in the hepatic lymph node of a Vaccinate. 

### 2.3. Culture

Four of eight Vaccinates and four of seven Controls had tissues that were culture positive for *M. bovis*. Vaccinates had a total of ten tissues that were positive on culture, three of which were not associated with gross lesions or microscopic lesions, representing two animals. Control animals had a total of eight positive tissues, one of which one was not associated with gross or microscopic lesions. Three Vaccinates had culture positive hepatic lymph nodes, whereas no Controls were culture positive in any abdominal tissues.

## 3. Discussion

Oral vaccination and boost by heat-inactivated *Mycobacterium bovis* shows moderate efficacy in controlling disease severity in wild pigs of Molokai genetics when experimentally infected with virulent *M. bovis* nineteen weeks after prime and twelve weeks after boost vaccination. This is especially evident when comparing the treatment groups in terms of gross and microscopic lesion scores of the thoracic region, as the lungs and associated lymph nodes of the Vaccinates sustained statistically lower lesion scores than did the Controls. Our observed outcomes are comparable to those produced in controlled studies in wild boar, in which significant protection against disease was demonstrated in animals vaccinated twice with inactivated *M. bovis* [13,14,15]. 

These results, although modest in effect, invite consideration of the vaccine having the ability of reducing the occurrence of airborne transmission of *M. bovis.* As summarized by Naranjo et al. in 2008 [16], lesion development in wild pig populations can vary; some populations experiencing lesions predominately in the head region, suggesting an oral route of infection, such as wild pigs in Australia in decades past. Other populations, such as wild boar in Mediterranean Spain, sustain more generalized disease, involving head and/or thorax, suggesting the possibility of multiple infection routes. We used an oral inoculation method for this study, because we believe that route likely predominates in wild pigs on Molokai. However, airborne transmission among pen mates may have occurred in our study as well. 

It is notable that three Controls and four Vaccinates did not sustain any lesions, nor were any of their tissues culture positive for *M. bovis*. It may be that these animals were not successfully colonized by the bacteria after administration of challenge material, or perhaps they were less susceptible to infection than their cohorts were. In this study, we did not perform additional assays that would indicate evidence of pathogen exposure, such as those that detect antibody development against *M. bovis* antigens. We therefore we could not determine if the animals effectively responded to infection and prevented colonization, or if no actual infection took place. Additional work is clearly needed to further evaluate the susceptibility of Molokai-origin pigs to *M. bovis* infection. 

It is interesting that, although not significant, Vaccinates did seem to have a higher rate of lesion development in the lymphoid tissues of the head region than did the Controls. In addition, three Vaccinates had positive cultures in hepatic lymph nodes, one of which also had a microscopic lesion, representing the only evidence of abdominal region infection in any of the animals in this study. Further studies exploring the pathogenesis of this strain of *M. bovis* in wild pigs from various geographic origins need to be performed to more fully understand these potential differences in response to and progression of infection. In line with these data, researchers in Spain have applied heat-inactivated *M. bovis* in free-ranging wild boar piglets with apparent success in reducing prevalence of disease caused by MTBC in vaccinated wild boar populations, compared to unvaccinated populations [8]. Should further investigation produce evidence that *M. bovis* persists in wild pig populations on Molokai Island, then consideration should be given to continuing research in the use of heat-inactivated *M. bovis* as part of an integrated disease management plan. 

Exploration of the feasibility of vaccination of wild pigs on Molokai Island against *M. bovis* infection must emphasize the importance of obtaining a comprehensive dataset on this population. Complete knowledge of the population size, distribution, behavior, habitat use, and disease status and ecology is crucial to targeting and applying disease management strategies most effectively. In addition, information on the disease status of other TB-susceptible species on the island must be obtained. This information is essential for development of effective vaccine delivery systems as well as developing metrics for measurement of vaccine success [17,18,19]. 

Future work specifically addressing vaccine performance should include additional controlled experiments with larger sample sizes. In tight isolation environments, logistical issues often limit our abilities to achieve desirable sample sizes, and more natural and minimally stressful conditions for these types of studies. Future work should also involve exploration of duration and nature of immunity, comparison with *M. bovis* BCG vaccination, and use of lower doses of challenge inocula, or a natural challenge model, to more closely represent transmission in the field. Again, up to date data need to be collected in order to determine if MTBC is still present and being maintained in the wild pig population of Molokai as well as in other wild animal populations on the island. If a reservoir situation is verified and characterized, further evaluation of disease management tools and strategies should be performed.

## 4. Materials and Methods 

### 4.1. Animals

Fifteen 3–4-month-old pigs were used for this study. Animals were offspring of wild pigs of Molokai, Hawaii, USA origin. The pigs were born at the United States Department of Agriculture, Animal and Plant Health Inspection Service/Colorado State University (CSU) Wildlife Research Facility (WRF), Fort Collins, Colorado, USA (40°34′5″ N, 105°08′49″ W; elevation approximately 1519 m). Pigs were housed for the vaccination portion of the study in two outdoor 3 m × 13 m solid panel runs with soil substrate, and were offered a commercial swine ration fed at 0.5 kg/animal/day and water ad libitum. For *M. bovis* challenge, the animals were transferred to the CSU, Biosafety Level 3 Animal Disease Laboratory (ADL), where they were housed in 3.7 m × 5.5 m rooms and were fed and watered as described above. This study was approved by CSU Institutional Animal Care and Use Committee (IACUC Protocol # 17-7486). 

### 4.2. Vaccination 

Heat-inactivated *M. bovis* strain (#1403, Neiker) was prepared by Neiker Tecnalia in Derio, Bizkaia, Spain as previously described [14,20]. The oral vaccine was shipped at a concentration of approximately 1 × 10^7^ colony forming units (cfu) killed *M. bovis* per 0.2 mL. 

Vaccine was administered orally within a candy mixed with cracked corn and fruit drink powder for flavoring. A 0.2 mL (1 × 10^7^ cfu) volume of vaccine or phosphate buffered saline (PBS) was pipetted into a 0.5 mL centrifuge tube. Each tube was embedded in a 2 cm bait made out of the fruit candy/cracked corn mix bait. 

Animals were randomly assigned with blocking for gender to two groups: Vaccinates (n = 8) or Controls (n = 7). On Day 0, pigs were anesthetized with isoflurane gas (Fluriso^TM^, Vetone, Boise, Idaho, USA) via mask, and identifying ear tags were administered to both ears. Following full recovery from anesthesia in individual recovery boxes, a bait with either vaccine or PBS was placed inside the recovery box and the pig was given time to eat the bait. Seven weeks after prime vaccination, each pig was boosted in the same manner as for the initial vaccination, without the anesthesia component. Animals were housed in mixed Vaccinate and Control groups until challenge.

### 4.3. Challenge 

Stock of *M. bovis,* strain 06-4387, originally obtained from a wild pig on Molokai, was received from the USDA APHIS Veterinary Services, National Veterinary Services Laboratory (NVSL), Ames, IA, USA and inoculated onto Middlebrook 7H11 agar plates. Cultures were incubated at 37 °C for 17 days, at which time confluent bacterial lawns had developed. Plates were flooded with PBS and bacteria were harvested with a swab. Those suspensions were sonicated briefly to break up clumps and counted using a Petroff–Hausser chamber. The resulting titer was 2.3 × 10^9^ bacteria/5 mL, and that suspension was diluted 1:2300 in PBS to yield an inoculum with a concentration of 10^6^/5 mL.

Fifteen weeks post-prime, all pigs were anesthetized with intramuscular (IM) midazolam, medetomidine, and butorphanol (Wildlife Pharmaceuticals Inc., Windsor, Colorado, USA) at 0.03, 0.06, and 0.03 mg/kg, respectively, for transportation to the ADL [21]. Animals were transported 0.2 km in a stock trailer and placed into two rooms in the same mixed groups as at the WRF. Anesthesia was reversed with atipamezole (Wildlife Pharmaceuticals Inc.) at 5 mg per 1 mg medetomidine administered. Nineteen weeks post-prime, pigs were sedated as above and orally challenged with 1 × 10^6^ cfu virulent *M. bovis* in 5mL PBS as described by Ballesteros et al. (2009) [22]. Anesthesia was reversed as described above.

### 4.4. Necropsy

Twelve weeks post-challenge, pigs were anesthetized with IM butorphanol, azaperone, and medetomidine (BAM^TM^, Wildlife Pharmaceuticals Inc.) at 0.027 mL/kg (0.73 mg/kg butorphanol, 0.24 mg/kg azaperone, and 0.27 mg/kg medetomidine), and euthanized via captive bolt [21]. The animals were necropsied and the following 23 tissues collected for gross examination, histopathology, and culture: lung (caudal (2), cranial (2), cardiac (2), and accessory lobe (1)), liver, spleen, kidney, mandibular lymph nodes (LN, 2), parotid LN (2), retropharyngeal LN (2), superficial cervical LN (2), tracheobronchial LN, hepatic LN, mesenteric LN, ileocecal LN, and tonsil. Any non-target tissues with apparent lesions were also collected. On gross examination, each tissue was assigned a lesion score as described by Garrido and others [20]. On microscopic examination, tissues were graded as follows: Grade 1: single or multiple small lesions, generally less than or equal to 1 mm, composed predominantly of inflammatory cells (PMNs, macrophages, and rare giant cells) usually having little or no central caseous necrosis and mineralization; Grade 2: single or multiple moderate-sized lesions, generally 2–3 mm, composed of inflammatory cells with moderate central necrosis and mineralization; and Grade 3: single or multiple large lesions, generally greater than or equal to 4 mm, containing inflammatory cells and marked central caseous necrosis and mineralization. Tissues for culture were placed in Whirl Pak^®^ bags (Nasco, Fort Atkinson, Wisconsin, USA) and stored at −70 °C until shipment to the NVSL for processing. Tissues for histopathology were placed in 10% formalin and sent to NVSL for processing. Fixed tissues were embedded in paraffin, sectioned at 5 µm, and stained with hematoxylin–eosin by use of standard procedures. Tissues with lesions compatible with tuberculosis underwent auramine-acridine orange and modified Ziehl–Neelson staining procedures (New Fuchsin) to detect the presence of acid-fast organisms.

### 4.5. Culture

Mycobacterial cultures were performed as previously described with some modifications [23]. Briefly, tissues were trimmed, homogenized in saline, and decontaminated with 4% NaOH for 10 min. Once samples were neutralized with a commercial buffer (IMMY, Norman OK, USA), they were centrifuged at 4600× *g* and the pellet was inoculated into both BACTEC MGIT media and 7H11 Middlebrook with 0.5% hemolyzed blood, 10% calf serum, 0.39% sodium pyruvate, and 0.025% malachite green as additives. Media were incubated according to manufacturer’s recommendations, and signal positive tubes were examined for the presence of acid-fast bacteria. If the media signaled positive prior to 42 days and no acid-fast organisms were detected, they were incubated at 37 °C for the full 42 days and screened by polymerase chain reaction (PCR) [20]. All PCR-positive MGIT media was subcultured onto solid media, and suspicious colonies were identified as *M. bovis* by PCR [24]. 

### 4.6. Statistics

Individual (not summed) gross and microscopic lesion scores within the treatment groups were compared using non-parametric Wilcoxon Signed Rank test (α = 0.05). Lesion scoring results in a set of scaled ordinal values and as such must be analyzed using methods that recognize the quantitative nature of the values but do not treat them as continuous numbers [25,26]. Although suitable parametric methods can be employed to analyze ordinal data, they operate best with large sample sizes [25,26]. The two treatment groups were limited to seven and eight individuals and we felt the best method for our dataset was non-parametric analysis. Data used for statistical analysis were comprised of all node pair scores in both Control and Vaccinate animals, where at least one lesion was detected in either treatment group. Nodes or node pairs where lesions were absent for both groups were not included as there were no lesion score data to compare. Table 1 shows which nodes or node pairs had lesion scores. We used a *Cohen’s d* test suitable for ordinal values to calculate effect size since sample size can confound resulting p-values [27,28]. All statistical analyses were carried out in R: A language and environment for statistical computing (Accessed 16 October 2019 and 1 March 2020).

## Figures and Tables

**Table 1:** Individual animal gross lesion scores, microscopic lesion scores, and *Mycobacterium bovis* culture outcome in tissues collected at necropsy from Vaccinates and Controls twelve weeks post-challenge with 10^4^ colony forming units (cfu) of virulent *M. bovis* strain 06-4387. Vaccinates received two oral doses of approximately 10^7^ cfu inactivated *M. bovis* at 19 and 12 weeks prior to challenge.

				Tissue (Gross Lesion Scores/Microscopic Lesion Score/*M. bovis* Culture Outcome: +/-)															
				Lung Lobe				Organ			Lymph Node/Lymphoid Tissue								
Treatment Group	Total Gross Lesion Score	Total Microscopic Lesion Score	ID	Caud^2,3^	Cran^2,4^	Card^2,5^	Acc^6^	Liver	Spleen	Kidney	Mand^2,7^	Parotid^2^	Retro-pharyngeal^2^	Superf Cerv^2,8^	Medias^9^	Tracheo-bronch^2,10^	Hep^11^	Mes^12^	Tonsil
Vaccinates	18	21	C	0/0/-	0/0/-	0/0/-	0/0/-	0/0/-	0/0/-	0/0/-	1,3/2,3/+	0/0/-	1,4/1,3/+	0/0/-	0/0/-	0/0/+	0/0/+	0/0/-	0/0/-
			D	0/0/-	0/0/-	0/0/-	0/0/-	0/0/-	0/0/-	0/0/-	0/0/-	4/3/+	0/0/+	0/0/-	0/0/-	0/2/+	0/0/+	0/0/-	0/0/-
			E	0/0/-	0/0/-	0/0/-	0/0/-	0/0/-	0/0/-	0/0/-	0/0/-	0/0/-	0/0/-	0/0/-	0/0/-	1/3/-	0/1/+	0/0/-	0/0/-
			H	0/0/-	0/0/-	0/0/-	0/0/-	0/0/-	0/0/-	0/0/-	0/0/-	0/0/-	0/0/-	0/0/-	0/0/-	0/0/-	0/0/-	0/0/-	0/0/-
			K	0/0/-	0/0/-	0/0/-	0/0/-	0/0/-	0/0/-	0/0/-	0/0/-	0/0/-	0/0/-	0/0/-	0/0/-	0/0/-	0/0/-	0/0/-	0/0/-
			L	0/0/-	0/0/-	0/0/-	0/0/-	0/0/-	0/0/-	0/0/-	0/0/-	0/0/-	0/0/-	0/0/-	0/0/-	0/0/-	0/0/-	0/0/-	0/0/-
			M	0/0/-	0/0/-	0/0/-	0/0/-	0/0/-	0/0/-	0/0/-	4/3/+	0/0/-	0/0/-	0/0/-	0/0/-	0/0/-	0/0/-	0/0/-	0/0/-
			N	0/0/-	0/0/-	0/0/-	0/0/-	0/0/-	0/0/-	0/0/-	0/0/-	0/0/-	0/0/-	0/0/-	0/0/-	0/0/-	0/0/-	0/0/-	0/0/-
Controls	36	33	A	0/0/-	0/0/-	0/0/-	0/0/-	0/0/-	0/0/-	0/0/-	0/0/-	0/0/-	0/0/-	0/0/-	0/0/-	0/0/-	0/0/-	0/0/-	0/0/-
			B	0/1/+^13^	0/0/-	0/0/-	0/0/-	0/0/-	0/0/-	0/0/-	4/3,1/+	0/0/-	0/1/-	0/0/-	0/0/-	0/2/-	0/0/-	0/0/-	0/0/-
			F	0/0/-	0/0/-	5/3,3/+	0/0/-	0/0/-	0/0/-	0/0/-	0/0/-	0/0/-	0/0/-	0/0/-	0/0/+	4,3/3,3/+	0/0/-	0/0/-	0/0/-
			G	0/0/-	0/0/-	0/0/-	0/0/-	0/0/-	0/0/-	0/0/-	0/0/-	0/0/-	0/0/-	0/0/-	0/0/-	0/0/-	0/0/-	0/0/-	0/0/-
			I	0/0/-	0/0/-	0/0/-	0/0/-	0/0/-	0/0/-	0/0/-	4/3/+	0/0/-	0/0/-	0/0/-	0/0/-	0/2/-	0/0/-	0/0/-	0/0/-
			J	0/0/-	0/0/-	5,5/2,3/+	0/0/-	0/0/-	0/0/-	0/0/-	0/0/-	0/0/-	0/0/-	0/0/-	0/0/-	3,3/3/+	0/0/-	0/0/-	0/0/-
			O	0/0/-	0/0/-	0/0/-	0/0/-	0/0/-	0/0/-	0/0/-	0/0/-	0/0/-	0/0/-	0/0/-	0/0/-	0/0/-	0/0/-	0/0/-	0/0/-

^1^ Average lesion score based on examination of 23 tissues; ^2^ Bilateral tissues were collected and total score from both tissues reported here; ^3^ Caud, Caudal Lung Lobes; ^4^ Cran, Cranial Lung Lobes; ^5^ Card, Cardiac Lung Lobes; ^6^ Acc, Accessory Lung Lobe; ^7^ Mand, Mandibular Lymph Nodes; ^8^ Superf Cerv, Superficial Cervical Lymph Nodes; ^9^ Medias, Mediastinal Lymph Node; ^10^ Tracheo-bronch, Tracheobronchial Lymph Nodes; ^11^ Hep, Hepatic Lymph Node; ^12^ Mes, Mesenteric Lymph Node; ^13^ Shaded cells have at least one positive outcome.
